# Is There Still a Place for Percutaneous Nephrolithotomy in Current Times?

**DOI:** 10.3390/jcm11175157

**Published:** 2022-08-31

**Authors:** Elisa De Lorenzis, Stefano Paolo Zanetti, Luca Boeri, Emanuele Montanari

**Affiliations:** 1 Department of Urology, Fondazione IRCCS Ca’ Granda Ospedale Maggiore Policlinico, Via della Commenda 15, 20122 Milan, Italy; 2 Department of Clinical Sciences and Community Health, University of Milan, 20122 Milan, Italy

**Keywords:** percutaneous nephrolithotomy, urolithiasis, endourology, kidney stones

## Abstract

Background: Percutaneous nephrolithotomy (PCNL) and its miniaturized modifications are usually considered the standard surgical options for large (>20 mm) staghorn and infected stones. Moreover, PCNL is a viable alternative to retrograde intrarenal surgery (RIRS) for smaller stones (<20 mm) in the presence of anatomical malformations or inaccessible lower pole stones. However, due to the advancements in laser and scope technology, RIRS is expanding its indications with the potential benefits of lower complications and a shorter hospital stay. Methods: A literature search using the PUBMED database from inception to June 2022 was performed to explore the current role of PCNL in endourology. The analysis involved a narrative synthesis. Results: PCNL confirmed its role in the treatment of large and complex stones; moreover, miniaturized PCNL has become more competitive, gaining space among classic indications of flexible ureteroscopy. Conclusions: considering all the evaluated subgroups, we can conclude that PCNL is an old fascinating procedure and is here to stay.

## 1. Introduction

The surgical treatment of urolithiasis has dramatically evolved over the last few decades. Nowadays, endourology (encompassing percutaneous and retrograde interventions) represents the solution to the vast majority of urinary stones.

The European Urology Guidelines [1] recommend percutaneous nephrolithotomy (PCNL) as the first-line option to treat kidney stones larger than 2 cm, followed by retrograde intrarenal surgery (RIRS) or extracorporeal shockwave lithotripsy (ESWL) as second-line treatments. For stones between 1 and 2 cm, ESWL and endourology are considered equally valuable options, while for stones smaller than 1 cm, PCNL is recommended only in case of failure or unfavorable conditions for RIRS of ESWL.

Since the first description of the techniques, both ureteroscopic and percutaneous instruments have greatly evolved, becoming more and more sophisticated and minimally invasive.

The history of ureteroscopy started in 1912 when Hugh Hampton Young [2] accidentally entered a dilated ureter with a pediatric cystoscope. Subsequently, longer and thinner instruments were produced to explore the ureter.

The game changer in the treatment of kidney stones was represented by the introduction of flexible ureterorenoscopes, which allowed for retrograde access to renal cavities. The progressive technological advancements in the visual systems, from fiber optics to digital video chips, the miniaturization of instruments, the evolution of the active and passive deflection mechanisms, and the development of sophisticated lithotripsy and lapaxy probes made flexible ureteroscopy a revolutionary technique that quickly changed the paradigm of urinary stones treatment. Endoscopic probes, such as laser fibers and baskets, have also greatly improved over time, making intracorporeal lithotripsy and lapaxy quicker and more efficient. To overcome the fragility and sterilization concerns of reusable flexible ureterorenoscopes, highly technological disposable instruments have been introduced in recent years.

The major limitations of RIRS are represented by the difficulties in retrieving a large number of fragments after lithotripsy of large stones and by the complicated balance between irrigation and intrarenal pressure. Indeed, the continuous rinse of renal cavities is needed to enhance visibility, but an unbalanced fluid evacuation may cause a pressure rise into the collecting system, with potential bacteria and toxins backflowing into the bloodstream and subsequent infectious complications [3].

For these reasons, in order to limit the operative time and prevent complications, large stones cannot be treated with a single RIRS procedure, but multiple sessions may be needed, exposing the patient to repeated anesthesia and the risk of ureteral damage and stenosis.

Percutaneous kidney surgery began to develop in 1954, when a radiologist first punctured a hydronephrotic renal pelvis to obtain a percutaneous pyelography [4].

In 1976, Johansson and Fernström first reported the extraction of kidney stones through a mature pre-existing percutaneous access under pure fluoroscopic control [5].

In the 1980s, the pioneers of PCNL around the world refined and described the percutaneous technique through a 22–26 Fr access, and applied in PCNL the concepts of visual endoscopic control and intracorporeal lithotripsy [6,7,8]. In 1998, the first series of mini-PERC in adult patients was published in order to reduce blood loss, pain, and hospital stay associated with standard PCNL [9]. A percutaneous 13-Fr ureteroscopy sheath was used with satisfactory results and a low complication rate. In the following years, mini-PCNL specific sets with 12–18 Fr access sheaths were manufactured and the technique further evolved thanks to the development of high power Holmium:YAG lasers for lithotripsy [10,11,12]. In 2011, the first micro-PCNL [13] with a 4.8 Fr all-seeing needle was performed, and in 2013, the ultra-mini-PCNL (UMP) [14] with an 11–13 Fr access diameter was described.

The main reported advantage of small caliber PCNL is the reduction in bleeding complications [15]. However, smaller instruments carry some limitations too, such as a reduced choice of lithotripsy and lapaxy probes, difficulties in fragment retrieval, longer operation time, reduced visual field, and higher intra renal pressure (IRP) [16]. In order to overcome these limitations, aspiration-integrated mini-PCNL systems have been developed in recent years, such as the Super Mini PCNL (SMP) and the vacuum-assisted mini-PCNL performed by means of the Clear Petra system [17].

Even if the technological evolution of flexible ureterorenoscopy made it gain popularity among endourologist also for stones larger than 2 cm, generally considering a prerogative of PCNL, other parameters beyond stone size need to be taken into account when establishing the best surgical approach. These are, for example, stone location, stone composition, pyelo-caliceal and renal anatomy, and the patient’s habitus.

In particular, the percutaneous technique still holds the primacy for the treatment of specific categories of stones and patients:

- staghorn stones
- infection stones
- stones in retrogradely inaccessible calices
- stones in patients with urinary diversions
- stones in patients with skeletal malformations
- stones in anomalous kidneys.

**Figure 1:**
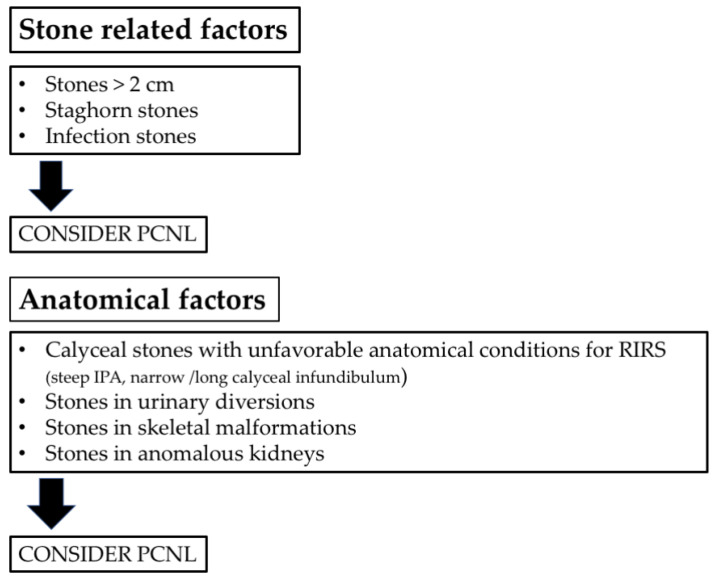
Flowchart for treatment planning (IPA = infundibular-pelvic angle).

Figure 1 summarizes a flow chart of treatment planning.

The aim of the current narrative review is to summarize and critically discuss the sempiternal and special indications of PCNL.

## 2. Materials and Methods

A literature search using the PUBMED database from inception to June 2022 was performed to explore classic and special indications of PCNL. Moreover, technological advancements in RIRS and PCNL were summarized. Keywords were “percutaneous nephrolithotomy”, “ureteroscopy”, “staghorn”, “lower pole stones”, “infected stones”, “skeletal malformations AND stones OR calculi”, “spinal deformity AND stones OR calculi”, “miniaturization”, “urinary diversions AND stones OR calculi”, “renal abnormalities AND stones OR calculi”. Boolean operators (AND, OR) were used to improve the search. In vitro and human articles were included. Articles identified through references were also included and analyzed. Original and review articles were included. After removing duplicates, animal models, and papers not relevant to the topic of this article, we identified 67 papers to be included in this narrative review.

The narrative review checklist is reported in Supplementary Figure S1.

## 3. Results

Results from the retrieved articles are presented in subparagraphs based on classic and special indications for PCNL.

### 3.1. Classic Indications of PCNL: Old but Gold

#### 3.1.1. Staghorn Stones

**Figure 2:**
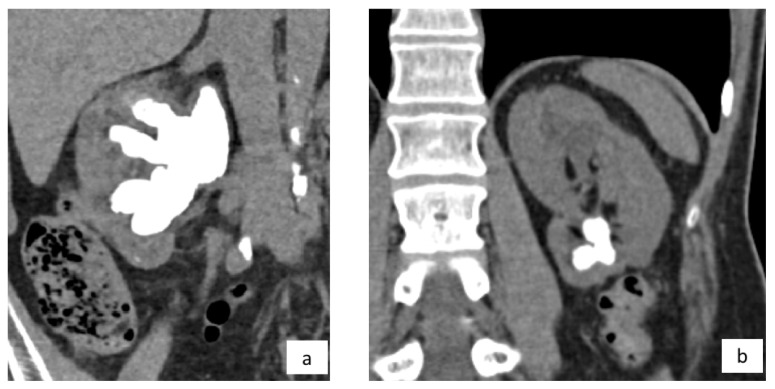
Staghorn calculi on CT scan. (**a**) Complete staghorn stone occupying the whole pyelocaliceal cavities; (**b**) Partial lower pole staghorn calculus.

Staghorn calculi are branched stones that involve a large part of the collecting system. Usually, they occupy the renal pelvis and branch into several or all of the calyces. There is no consensus regarding the definition of staghorn calculi or a volume criteria to divide staghorn stones into “partial” or “complete”. Typically, a complete staghorn calculus involves all the collecting systems. Meanwhile, a partial staghorn stone occupies only a portion of the renal cavities (e.g., at least 2 calyces) [18]. Two examples of staghorn calculi are depicted in Figure 2.

Staghorn calculi are most frequently composed of magnesium ammonium phosphate and/or calcium carbonate apatite. Additionally, cystine and uric acid stones can grow in a staghorn structure; calcium oxalate or phosphate stones have a less frequent branched growth [19].

The goals of the treatment of staghorn stones are complete stone clearance, resolution of the obstruction, and restoration of kidney function with minimal morbidity.

PCNL is historically considered the standard of care for the treatment of staghorn calculi [18]. Usually, ureteroscopic management of these calculi is considered inferior to PCNL due to its low stone-free rates (SFRs) and the need for multiple sessions [19,20,21]. A recent meta-analysis explored the integration of ureteroscopy with PCNL in a combined antegrade and retrograde approach for the treatment of staghorn calculi [22]. The authors defined five advantages of this synergistic approach. First, an initial retrograde lithotripsy can facilitate the percutaneous placement of the guidewire and the tract dilation [23]. Second, the staghorn stone can be managed simultaneously by the nephroscope and the ureteroscope, saving time. Third, in the case of stones allocated in an angulated calyx, the access can be easier for the ureteroscope. Forth, the ureteroscope can be used to displace the stone in a more convenient location for the nephroscope, the so-called “pass-the-ball” technique [24]. The last advantage is the use of the flexible ureteroscope to explore all the renal cavities and the ureter to assess the final clearance status.

Even if PCNL has become the most commonly used first-line treatment for large and complex renal stones, the management of this type of calculi is still one of the biggest challenge in PCNL, with potentially higher rates of complications and lower SFR.

A 2011 study from CROES found that postoperative fever, bleeding, and blood transfusions were more common in the case of staghorn stones, and median operative time and hospital stay were longer [25]. Moreover, comparing patients with staghorn stones to patients with nonstaghorn stones, the SFR was lower (56.9% vs. 82.5%) and multiple punctures were more frequently employed (16.9% vs. 5.0%) in case of branched calculi.

In the case of staghorn and large-volume renal stones, the debate revolves around the use of single or multiple tracts during PCNL. The choice should be taken balancing morbidity and stone clearance. Theoretically, as the number of tracts increases, the blood loss might increase.

A multitract PCNL can be planned because a single tract cannot be the optimum access for treating all the caliceal branches. This procedure has been demonstrated to be safe and feasible in expert hands and is associated with higher stone clearance than single tract PCNL [26]. The percentage of renal functional loss is similar in multiple versus single tracts, but the bleeding complications are more frequent in multitract PCNL [26,27,28,29,30].

In order to reduce the morbidity of a multitract PCNL, a small access sheath and a flexible scope can be employed, and the procedure can be staged in different sessions [31,32]. Moreover, complications can be reduced by taking preventive measures, such as ultrasound-guided punctures and limiting the lithotripsy time to 90 min [33].

Another technique described in the context of multitract PCNL is the combination of a standard-tract (24 Fr) with a mini-tract (16 Fr) in the same session [34]. Compared to single standard-tract PCNL, standard-tract combined with mini-tract PCNL is associated with similar transfusion rates, postoperative Clavien score, and operative time, but had significantly higher SFR and lower rates of second PCNL.

More recently, even in the case of complex staghorn stones, encouraging results of single-access PCNL were reported. A multi-institutional study demonstrated a mean operative time of 80.1 min, an overall complication rate of 18% (3.7% of Clavien-Dindo grade ≥ 3) and a transfusion rate of 0.7%. Only in 18.2% of the cases, fragments ≥ 4 mm were found after primary single-access PCNL [35].

The outcomes of the PCNL may be affected not only by the stone volume, but also by the morphometry of the staghorn stones and by the renal anatomy.

The study of the morphology of the pelvicalyceal system is essential in order to plan the proper type of treatment and the surgical strategy. For example, in the case of a wide collecting system with a broad calyceal infundibula, the exploration of all the calyces can be performed using a single percutaneous tract. The presence of a large stone burden involving an unfavorable calyx can require an additional percutaneous tract for its clearance.

The need for torquing the rigid nephroscope inside the excretory system to reach an inaccessible calyx can determine extravasation and bleeding.

A study by Mishra et al. [36] developed a computed tomography (CT) urography staghorn morphometry-based prediction algorithm to foresee the number of tracts and stages for PCNL. The created model showed that a single-tract single-stage PCNL is more likely for calculi with an overall volume smaller than 5000 mm^3^ and with a small amount of stone inside an unfavorable calyx (<5%).

Regarding the patient position, prone and supine PCNL are both feasible for the management of staghorn stones, although the upper pole access is more common in the prone approach. However, the final decision on the patient’s position depends on the surgeon’s preference and experience.

With the advent of minimally invasive techniques, laparoscopic and robotic pyelolithotomy, or anatrophic nephrolithotomy, were considered as alternatives to PCNL or open surgery in the treatment of large and/or complex renal stones. Usually, surgery is preferred when lithiasis is associated with an anatomic anomaly, such as a ureteropelvic junction obstruction or a large calyceal diverticulum.

A prospective randomized comparison of open surgery versus PCNL for complete staghorn stones demonstrated that PCNL is associated with shorter operative time and hospital stay, faster return to work, and lower intraoperative complications. Postoperative complications, stone clearance, and improvement in renal function were similar among groups [37].

An updated meta-analysis compared laparoscopic pyelolithotomy and PCNL for the management of large renal pelvic stones [38]. Laparoscopic pyelolithotomy had a higher SFR, lower blood transfusion rate, less bleeding, less postoperative fever, and lower retreatment rate. PCNL was associated with shorter operative times and hospital stay. The authors concluded that PCNL is still suitable for most cases, and laparoscopic pyelolithotomy can be used as an alternative procedure in selected cases.

#### 3.1.2. Infection Stones

The relationship between infections and renal stones is well known. Magnesium ammonium phosphate (struvite) and triple phosphate renal calculi develop as a result of urinary tract infection by urease-producing pathogens, including both gram-positive and gram-negative species (such as Proteus, Staphylococcus, Pseudomonas, Providencia, and Klebsiella).

Struvite stones represent 2–15% of the stones sent for analysis [1].

Infection is one of the most common etiologies of staghorn stones, which, in these cases, can grow rapidly (within 4–6 weeks), occupying the entire collecting system.

Apart from being responsible for the stone composition, bacteria can also colonize other types of stones [39]. The term “infection stones”, or better “infected stones”, can also be related to this type of calculus, in this case, also including calcium oxalate and calcium phosphate ones.

There is no gold standard method to diagnose struvite stones preoperatively; they can be suspected in the presence of urease-producing bacteria in midstream culture, development of staghorn growth in a short time, and low and heterogenous Hounsfield unit value on a CT.

The risk factors for the development of struvite stones are female gender, extreme ages, diabetes mellitus, urinary tract malformations/obstructions, urinary diversion, neurogenic bladder, urinary stasis, and indwelling urinary catheters [40].

The therapy of struvite stones is mainly based on three pillars: (1) complete eradication of stone fragments; (2) initial antibiotic therapy and maintenance of sterile urine without antibiotics; and (3) prevention of recurrence [41].

The complete clearance of all stone material is crucial because remaining fragments can serve as a nidus for new stone formation, also considering the rapid growth of these stones. Recurrence rates are very high if residual fragments remain in place [42].

Antibiotics may suppress bacteriuria, but rarely totally eliminate an infection in the presence of stones.

The gold-standard approach for infection stone removal is PCNL. In rare cases, when this approach is not viable, such as a pelvic kidney, retrorenal colon, or spinal deformities that make the percutaneous access to the kidney impossible, anatrophic nephrolithotomy might be considered.

ESWL has a limited role for staghorn infection calculi due to its low efficacy in the treatment of large stone burdens. Moreover, in this subset of stones, ESWL can be associated with potential complications, including sepsis, perinephric haematoma, and obstructive nephropathy from steinstrasse.

Considering that most of the time, staghorn stones are composed by struvite, the surgical management of magnesium ammonium phosphate stones is usually similar to that of staghorn stones as previously described.

Retrograde flexible ureteroscopy, accessory mini-PCNL tracts, and flexible nephroscopy can be used in combination with PCNL in the treatment of staghorn infection stones, as valid adjuncts to PCNL in achieving optimal SFR.

Due to the presence of bacteria, infectious complications can develop after infection stone treatment, although adequate pre-operative antibiotic therapy.

A recent study identified some predictors of urosepsis after PCNL treatment for struvite stones: higher preoperative white blood cell count, greater total stone surface area, preoperative stenting, recent use of antibiotics, and obstructive uropathy [43]. In another study, sepsis was approximately four times more common in patients with struvite stones treated with PCNL. In multivariate analysis, the preoperative presence of multidrug resistant bacteriuria and increased serum creatinine were independent risk predictors of sepsis [44].

#### 3.1.3. Lower Pole Inaccessible Calices and the Matryoshka Technique

Lower pole stones may remain asymptomatic in many patients; however, the treatment of these calculi is challenging due to their anatomically complex access to the inferior renal calyx and the potential difficulties in eliminating fragments after lithotripsy.

Regardless of the stone size, in case of unfavorable anatomical conditions, such as long calyx, narrow infundibulum, and steep infundibular-pelvic angle (IPA), PCNL can be an option for lower pole calculi [1].

In these conditions, the efficacy of ESWL is limited. For example, in the presence of IPA less than 90 degrees, infundibular width ≤ 5 mm and infundibular length > 3 cm, the ESWL success rate is only 17% [45].

Sampaio et al., proposed a technique to measure the IPA [46]. The infundibulo-pelvic angle is measured from the intersection of two lines. The first line links the central axis of the proximal ureter with the central axis of the ureteropelvic junction, while the second line is parallel to the orientation of the main infundibulum in the lower calyx where the stone is localized.

The IPA can also affect the success of flexible ureteroscopy by preventing access to the lower pole despite the complete deflection of the scope. Moreover, the use of accessory instruments (laser fiber, basket) may further reduce this deflection.

Of note, a study evaluating the incidence of damage in reusable flexible ureterorenoscopes found that 31/32 damaged instruments were used to explore the lower pole [47]. The authors also demonstrated that laser lithotripsy of multiple and large stones in the lower kidney pole and a steep IPA (≤50°) are the main risk factors for flexible ureterorenoscope damage.

Geavlete et al., concluded that an acute IPA (<30°) or the association between a long infundibulum (>3 cm) and a reduced angle may have a significant impact over ureteroscopy success [48].

A more recent study confirmed that an IPA lower than 90 degrees, larger stone burden, and lower pole stones are statistically significantly associated with residual stone fragments and need for repeated surgery after retrograde flexible ureteroscopy [49].

Advancements in technology have improved the deflection of the scopes, however, in cases with negative anatomic predicting factors and medium stone burden, other approaches, such as miniaturized PCNL, should be considered. A recent meta-analysis compared mini-PCNL (diameter between 14 and 22 Fr) and RIRS for treatment of 10–20 mm lower pole stones [50]. Mini-PCNL was associated with a higher success rate, but with a similar safety profile and no difference in fluoroscopy and operative time, hospitalization, and overall complication rate compared with RIRS.

Another example of a surgical treatment tailored to the characteristics of both the patient’s anatomy and the stone is the so-called Matryoshka technique [51]. This strategy allows us to dynamically and intraoperatively adapt the tract size to the anatomy of the pyelocalyceal system. Some indications for this approach include: a calyceal staghorn stone, a narrow infundibulum containing the stone, or the impossibility of advancing the guidewire towards the renal pelvis and the ureter after puncturing the calyx. This strategy implies the initial adoption of a small size tract in order to perform minimal lithotripsy, which creates enough space to insert a safety guidewire into the system. After this, with a stabilized access, the anatomical relationship between the calyx and the stone is evaluated, and it is possible to proceed with an adequate and safe tract dilation.

### 3.2. Special Indications of PCNL

#### 3.2.1. Skeletal Malformations

**Figure 3:**
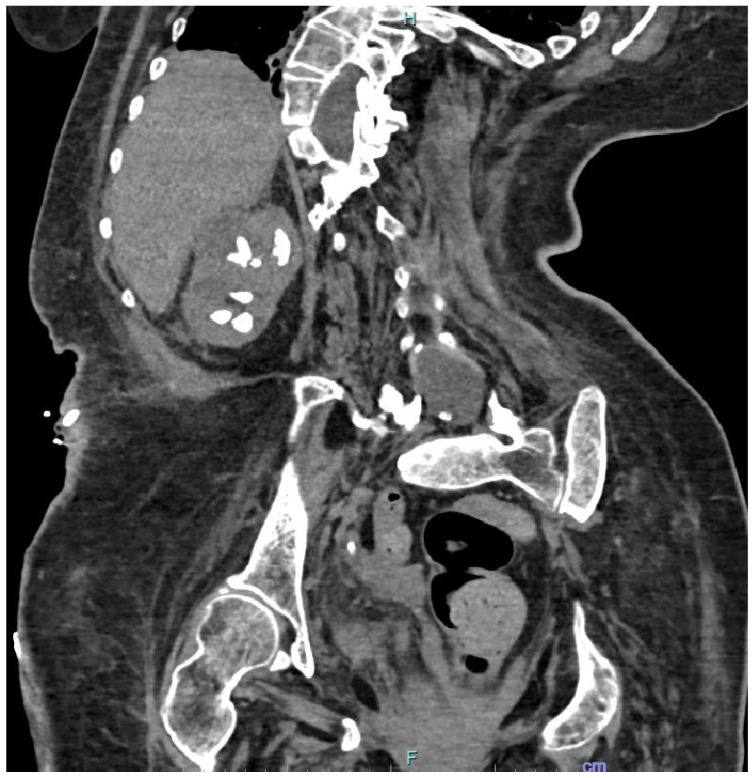
CT scan showing multiple kidney stones in a patient affected by spina bifida.

Spinal deformity (scoliosis, kyphosis, or kyphoscoliosis) is caused by the pathologic curvature of the thoracic and/or lumbar regions of the spine and it is usually associated with systemic involvement, including cardiac, genitourinary, pulmonary, and neurologic anomalies [52]. It is estimated that scoliosis affects 2% of women and 0.5% of men in the general population, although rates may vary quite significantly based on the specific definition of scoliosis and which patient population is being studied [53]. Urolithiasis is a common condition in patients with spinal deformity and may be caused by immobilisation, voiding dysfunction, metabolic disorders, such as hypercalcaemia, and chronic urinary infections [54] (Figure 3). It is reported that the risk of a urinary stone disease in these patients is up to 20% [54,55].

The treatment of renal stones in such patients is complex; the indications are not clear and are based on personal experience. Issues, such as respiratory dysfunction, difficulty in positioning and stabilizing the patients, and profound anatomic variations are the main contributors to the complexity.

The vast majority of stones in this population are soft and radio-opaque. Therefore, one can recommend ESWL as a first-line treatment option if there are no medical contraindications. However, ESWL might be technically challenging and have poor outcomes in this patient population since proper positioning is hard to achieve, making wave focus on target difficult [56]. Post-ESWL fragment passage can be hindered by aberrant renal locations, and the risk of renal parenchymal and vascular damage developing after ESWL might be higher due to issues in patients’ positioning [57].

Ureteroscopy (URS) has difficult restrictions due to anatomic variations and the high stone burden typical of these patients might require multiple procedures [58]. SFRs of 35.7–75% and a 40% complication rate were described in several publications [59,60].

PCNL has gradually emerged as the preferred treatment option for kidney stones in patients with skeletal anomalies. Nonetheless, several issues should be addressed before undertaking percutaneous surgery in this population.

Severe scoliosis may lead to thoracic or pelvic deformity, thus, altering the internal anatomy, and increasing the risk of injuring neighboring organs during PCNL. The impact of scoliosis on the human body can restrict lung ventilation and consequently result in respiratory dysfunction. The type of anesthesia and patient positioning during PCNL have to be evaluated carefully. The supine position of the patient during PCNL allows for better airway control and improved ventilation than the prone one [53,61]. Moreover, retrograde ureteroscopy can be used in combination with PCNL in the supine position if needed. However, previous reports have shown that surgery in the prone position is as equally safe and effective as in the supine or lateral positions, with the advantage of providing enough space for access establishment and for the movements of the endoscopic instruments [53,62]. The key determinant of the complexity of the operation is the relative locations of the affected kidneys, the curved spine, and the surrounding organs. If the spine is convex to the ipsilateral side of the renal stone, it is easy to get a satisfactory position and percutaneous access to the kidney, which would then be easily exposed. On the contrary, if the target kidney is on the concave side of a curved spine, it can result in a difficult puncture and dilatation. The difficulty of access establishment and lithotripsy is due to the limited room between the thorax and the pelvis, and the kidney being squeezed by the internal organs.

Puncture can be per-formed under the guidance of ultrasound (US), fluoroscopy, or CT. US can accurately observe the structure of the kidney, improve the visualization of adjacent viscera, clearly delineates the anterior and posterior calyces, and avoids vascular injury with doppler flow imaging. Access for PCNL using conventional fluoroscopic guidance may carry an increased risk of damage to surrounding organs in patients with renal calculi and aberrant anatomy. The US-guided fluoroscopic adjusted puncture through the cup of the desired calix offers many advantages and it is one of the preferred methods of kidney access in these situations. CT guidance offers cross-sectional imaging with precise anatomic details; but it requires a two-step procedure, exposes the patient and the physician to more radiation, and depends on a radiologist being present.

In terms of clinical outcomes, few reports have described the safety and efficacy of PCNL in patients with spinal deformity. Montanari et al. investigated eight patients with spinal deformities who underwent supine (*n* = 5) or prone (*n* = 5) PCNL [53]. They found an overall SFR of 88.8%, but 40% complication rate with 20% of patients needing blood transfusion, and 10% experiencing major complications. No injuries of surrounding organs or anesthesia-related complications were reported.

A similar SFR of 93% was found in a recent study with 16 patients with spinal deformities treated with prone PCNL [63]. The authors reported complications in 31.2% of cases, thus, including 12.5% of transfusions and 6% of major complications. Symons et al., reported 39 PCNLs on 29 patients with spinal deformity including 10 patients with spina bifida. They experienced two postoperative deaths and three major complications including seizure, aspiration pneumonia, and pressure necrosis [64]. Lastly, Wang et al. analyzed data from 84 PCNL performed in 72 patients with spinal deformity [61]. They reported a SFR of 89.9% with four (5%) patients requiring blood transfusion and 20 (23.8%) participants with postoperative fever. As a whole, published data suggest that PCNL in this group of patients is challenging but effective, also considering staged or additional procedures to achieve the stone free status, with slightly higher rate of postoperative complications. This is mostly related to the difficulty of kidney puncture and the higher risk of postoperative infectious complications of this cohort.

#### 3.2.2. Stones in Urinary Diversions

Several factors contribute to stone formation in patients with urinary diversion, thus, including urinary stasis, mucus formation and inadequate clearance, bacterial colonization, outlet obstruction, and foreign bodies, such as staples and nonabsorbable sutures used to reconstruct the lower urinary tract [65]. Concerning the upper urinary tract, common etiological causes are mucus reflux into the upper tract and ureteral or uretero-enteric strictures. Furthermore, urinary diversions often utilize colonic or ileal segments, which results in hyperchloremic metabolic acidosis that predispose to stone formation. Retrograde ureteroscopy in patients with urinary diversion can be technically difficult as the ureteral orifice may be hard to identify and navigation in tortuous and dilated ureters may be necessary. SFRs reported in the literature are inferior compared to series of patients with normal anatomy [66,67].

For these reasons, PCNL is often the preferred treatment option in patients with urinary diversion.

Zhong et al., analyzed a series of 26 patient with urinary stones after cystectomy and urinary diversion [68]. PCNL was performed in 19 patients with kidney/upper ureter stones with a SFR of 89.5%. Only 2 (7.7%) patients experienced postoperative complications. Of note, pure RIRS was firstly attempted in four patients, but all failed due to difficulty in entering the uretero-enteric anastomosis. Therefore, anterograde flexible ureteroscopy was performed through a percutaneous access. In a similar study, PCNL and antegrade URS were performed in 20 and 4 patients after cystectomy and urinary diversion. Overall SFR was 87.5% and complications were reported in only 12.5% of participants [67].

In conclusion, PCNL is a safe and effective treatment option for kidney stone in this population; moreover, an antegrade approach should also be considered after retrograde failure for middle and lower ureteric stones.

#### 3.2.3. Renal Abnormalities

Anomalous kidneys arise from different abnormalities in the embryological development: abnormal ascent, fusion, rotation, or a combination of these. Anatomical anomalies not only lead to compromised renal drainage, but also increase the risk of urolithiasis [69].

The choice of the procedure for stone treatment must take into account the difficult access associated with the anatomical positioning of the kidney, the location of the stone, the individual surgeon’s skill, and available equipment.

Horseshoe kidney (HSK) is the most common renal fusion anomaly [70]. Anterior displacement of the renal pelvis and high insertion of the involved ureter cause urinary abnormal drainage with flow hinderance and urinary stasis in the collecting system which may result in stone formation. The reported incidence of urolithiasis in patients with HSK varies between 20% to 60% in different series [71]. ESWL, URS, and PCNL are all potential treatment options for stones in patients with HSK. Previous studies have shown that SFR after ESWL in this population was variable (31–100%), but the main disadvantage was the anatomic abnormalities that may prevent fragment passage in a substantial number of the cases [72].

PCNL and URS have been widely investigated for the treatment of kidney stones in the HSK population. Kartal et al. investigated 49 patients with HSK submitted to URS (*n* = 29) and PCNL (*n* = 21) [73]. A single session and final SRF were registered in 71.4% and 85.7% in URS, and 81% and 90.5% in PCNL groups, respectively (all *p* > 0.05). URS group showed shorter hospital stay but higher retreatment rate. Importantly, postoperative complications were similar among the groups. A similar study compared outcomes from 50 and 38 patients with HSK treated with URS and PCNL between 2007 and 2016 [71]. The authors reported a similar SFR after URS (82%) and PCNL (84.2%) but more sessions of retrograde surgeries were necessary to achieve the result. No difference was noted in terms of postoperative complications. More recently, Vicentini et al. performed a multicenter study analyzing 106 PCNL in HSK patients and showed that the SFRs and complications rates were similar to those from surgeries in patients without renal anomalies [74]. The supine approach led to similar outcomes compared to the prone one.

In clinical practice, the deflection and handling of the flexible ureteroscope are made more difficult, as patients with HSK have flatter pelvises and narrower intrarenal spaces. The abnormal structure of the kidneys, the high insertion of the ureters, and a narrow infundibulo-pelvic angle make the procedure more difficult, the SFRs lower, and increase the probability of a second session. Additionally, stone passage is more difficult in HSK patients, therefore, stone fragmentation during URS should be as complete as possible, thus increasing the operative time and the risk of bleeding and infectious complications. Taken together, results suggest that PCNL is comparable to URS in terms of SFR and complications in HSK patients but with lower need for additional procedures.

The pelvic kidney occurs owing to a failure of its ascent during development that makes it stay below the pelvic brim, laying over the sacrum and caudal to the aortic bifurcation [75]. Generally, pelvic kidneys have a high insertion of the ureter and are malrotated, predisposing to urinary stasis and nephrolithiasis. As a consequence, the approach to the pelvic kidney is a big challenge. Few studies described URS in pelvic kidney with a SFR ranging 75–85%, but all declared extreme difficulty in achieving the pelvicalyceal system through the tortuous ureter [76].

PCNL is the most common approach for the management of large stone burden, and in pelvic kidneys it has proved to be as effective as in normal kidneys [77]. Otano et al. analyzed 26 patients with ectopic pelvic kidneys treated with PCNL and reported 88% SFR, with few rates of complications [78].

Overall, the literature shows that PCNL is feasible in ectopic pelvic kidney treatment, with similar outcomes compared to patients with normal anatomy.

Stones can become a complication in patients after kidney transplant. The presentation of stone disease in transplanted kidneys is often late as they are denervated and so pain is not a typical feature of obstruction. Fever, hematuria, and worsening renal function are not uncommon presentations [75]. URS is often technically difficult due to the angle and position of the ureteroneocystostomy at the dome/anterior wall of the bladder and stone-free rates of 60–70% are reported in small case series [79,80]. PCNL is facilitated by the anterior position of the allograft kidney using ultrasound-guided or CT-guided puncture [81]. Antegrade access with a flexible ureteroscope is an option for managing ureteric stones to limit the dilatation required [82]. Previous studies have reported high SFRs (66–100%) with PCNL when combined with the use of flexible instrumentation and baskets in this population [83,84,85].

In Table 1 we summarize treatment results for specific categories of stones and patients as reported in the literature.

## 4. Discussion

As previously mentioned, the technological evolution of retrograde endoscopic instruments has led to a progressive gain in popularity of RIRS, also for stones larger than 2 cm. A systematic review and meta-analysis by Aboumarzouk and colleagues examined this topic, demonstrating a mean SFR of 93.7% (77–96.7%) with a good safety profile for stones with a mean size of 2.5 cm [86]. However, the average number of procedures per patient was 1.6 and all the studies included in the meta-analysis were from high-volume experienced centers; subsequently, the reported results may not be reproducible in every institution. Moreover, a study by Erkoc et al. compared mini-PCNL and RIRS for stones between 2 and 3 cm, demonstrating a significantly higher SFR for mini-PCNL with respect to RIRS after a single procedure [87]. Concerning safety, in this study, mini-PCNL was characterized by higher analgesic requirements and hemoglobin loss, but without an augmented need for transfusions nor higher sepsis or complication rates.

On the other hand, the miniaturization of percutaneous scopes and access sheaths, combined with the recent introduction of aspirating sheaths, has made PCNL less invasive and more efficient, and also attractive for smaller stones. Jia et al. reported a better SFR, a lower retreatment rate, and a lower complication rate for SMP with respect to RIRS for pediatric upper urinary tract stones between 1 and 2 cm [88]. In a similar stone size range (<2 cm), a randomized controlled study demonstrated that SMP is equally effective but safer than standard PCNL [89]. Indeed, albeit characterized by longer operative time, SMP is associated with lower bleeding, lower postoperative pain, and a shorter hospital stay.

In terms of PCNL evolution, the thulium fiber laser (TFL) is the latest laser technology introduced. Compared to the classic Ho:YAG laser, the TFL is characterized by a smaller fiber diameter, reduced retropulsion, higher frequency, and shorter lithotripsy time with minimal collateral tissue damage [90,91]. Therefore, its use has gained increased attention for kidney stone treatment in clinical practice. Enikeev et al. reported the first use of the TFL in mini-PCNL in a cohort of 120 patients [92]. The mean operative and laser on time were 23 and 5 min, respectively. The overall SFR was 85%, but a 17% rate of complications was noted. Subsequently, the same group of authors investigated the outcomes of PCNL with TFL used in different settings among 125 patients [93]. The overall SFR was 85% and minimal complications were reported, all not related to the laser lithotripsy. Of note, surgeons reported minimal and absent retropulsion, along with better visibility in high-frequency regimens. Shah et al. analyzed a cohort of 54 patients treated with mini-PCNL with TFL and aspiration-assisted nephrostomy sheath [94]. The authors reported 100% SFR and only three patients had Clavien II complications. More recently, Patil et al. compared outcomes of mini-PCNL performed with TFL or Trilogy™ in a nonrandomized study [95]. Preoperative patients and stone characteristics were comparable among 30 participants in both groups. The authors reported that SFR and postoperative complications were similar among groups. In summary, data from preclinical and clinical studies showed that TFL has the potential to compete and be considered an alternative to the Ho:YAG laser. Its safety and efficacy, combined with the reduced retropulsion effect, make TFL a game changer for stone treatment. However, large multicenter, prospective, and randomized clinical trials with a Ho:YAG comparison are needed to confirm the aforementioned observations.

In conclusion, apart from the classic indications (staghorn and infectious stones, stones in retrogradely inaccessible calyces, stones in urinary diversions, skeletal deformities, and anomalous kidneys) for which PCNL is far from being ruled out, we are assisting to an overlap of the indication of miniaturized PCNL and flexible ureteroscopy, which became an alternative, in some cases, according to the surgeon’s preference.

This means that, in current times, not only is there still a place for percutaneous nephrolithotomy, but its application, in specialized centers, is even growing at the expense of RIRS.

## Figures and Tables

**Table 1:** Treatment results for specific categories of stones and patients as reported in the literature.

Stone Category	Study and Design	Success Rate	Complication Rate
**Staghorn stones**	Desai et al., 2011 [25]; prospective cohort	56.9%	Not reported as overall datum, but a higher rate than in the case of the nonstaghorn stones
	Large et al., 2021 [35]; retrospective review	81.8%	18%
	Al-Kohlany et al., 2005 [37]; prospective randomized study	74.4%	16.6%
	Bai et al., 2017 [38]; meta-analysis	87.92%	Not reported as overall datum, but a lower rate of single complications after PCNL than after laparoscopic pyelolithotomy
**Stones in calyces with difficult retrograde access**	Cabrera et al., 2020 [50]; meta-analysis	Higher stone-free rate after PCNL than after RIRS for lower pole stones	No significant differences in the complication rate after PCNL than after RIRS for lower pole stones
	Zanetti et al., 2021 [51]; retrospective study	62.5%	25%
**Stones in patients with skeletal malformations**	Montanari et al., 2010 [53]; retrospective study	88.8%	40%
	Izol et al., 2015 [63]; retrospective study	93%	31.2%
	Symons et al., 2006 [64]; retrospective study	62%	35.9%
	Wang et al., 2019 [61]; retrospective study	89.9%	36.1%
**Stones in patients with urinary diversions**	Zhong et al., 2018 [68]; retrospective study	89.5%	7.7%
	el-Nahas et al., 2006 [67]; retrospective study	87.5%	12.5%
**Stones in horseshoe kidneys**	Kartal et al., 2019 [73]; retrospective study	90.5%	38.1%
	Eryildirim et al., 2018 [71]; retrospective study	84.2%	50%
	Vicentini et al., 2021 [74]; retrospective study	54.7%	17.5%
**Stones in pelvic kidneys**	Otaño et al., 2015 [78]; retrospective study	88%	23.1%

## Data Availability

Not applicable.

## Supplementary Materials

The following supporting information can be downloaded at: https://www.mdpi.com/article/10.3390/jcm11175157/s1, Figure S1: narrative review checklist. Ref. [96] is cited in Supplementary Materials.
