# Supplementary material for: Is There Still a Place for Percutaneous Nephrolithotomy in Current Times?
Source: J Clin Med. 2022 Aug 31;11(17):5157. doi: 10.3390/jcm11175157 (PMC9457409; doi:10.3390/jcm11175157)
Supplement: Supplementary file 1 [file jcm-11-05157-s001.zip › jcm-1856538-supplementary.pdf]

Narrative Review Checklist

Section/Topic	Item No	Item	Reported on Page Number/Line Number	Reported on Section/Paragraph
**TITLE**				
Title	1	Identify the report as a Narrative Review or Literature Review.	1	Title
**ABSTRACT**				
Structured summary	2	Provide a structured summary with the subsections as: objective, background, methods, conclusion (1).	Abstract	Abstract
**INTRODUCTION**				
Rationale/background	3	Describe the rationale for the review in the context of what is already known.	1-3	Introduction
Objectives	4	Specify the key question(s) identified for the review topic.	3	Objectives
**METHODS**				
Research selection	5	Specify the process for identifying the literature search (eg, years considered, language, publication status, study design, and databases of coverage).	3	Methods
**DISCUSSION/SUMMARY**				
Narrative	6	Discuss: 1) research reviewed including fundamental or key findings, 2) limitations and/or quality of research reviewed, and 3) need for future research.	3-11	Results-Discussion
Summary	7	Provide an overall interpretation of the narrative review in the context of clinical practice and/or the Nutrition Care Process for registered dietitian nutritionists, clinical practice for other health professionals, policy development and implementation, or future research.	12	Discussion

Figure S1. narrative review checklist [1].

1. Green, B.N.; Johnson, C.D.; Adams, A. Writing narrative literature reviews for peer-reviewed journals: Secrets of the trade. *J. Sports Chiropr. Rehabil.* **2001**, *15*, 5–19.
